# Prevalence of haemotropic mycoplasmas and blood piroplasmids in domestic and wild ruminants in Slovakia, Central Europe

**DOI:** 10.1016/j.crpvbd.2025.100270

**Published:** 2025-05-15

**Authors:** Dana Zubriková, Lucia Vargová, Júlia Halapy, Branislav Lukáč, Veronika Blažeková, Klaudia Mária Švirlochová, Eva Čisovská Bazsalovicsová, Ján Čurlík, Ivana Heglasová, Bronislava Víchová

**Affiliations:** a Institute of Parasitology, Slovak Academy of Sciences, Hlinkova 3, Košice, 041 81, Slovakia; b Small Animal Clinic, University of Veterinary Medicine and Pharmacy, Komenského 73, Košice, 041 81, Slovakia; c Department of Epizootiology, Parasitology and Protection of One Health, University of Veterinary Medicine and Pharmacy in Košice, Komenského 73, Košice, 041 81, Slovakia; d Department of Breeding and Diseases of Game, Fish and Bees, Ecology and Cynology, University of Veterinary Medicine and Pharmacy in Košice, Komenského 73, Košice, 041 81, Slovakia; e Department of Biology and Environmental Studies, Faculty of Natural Sciences, Matej Bel University, Tajovského 40, 974 01, Banská Bystrica, Slovakia

**Keywords:** Haemotropic *Mycoplasma*, *Babesia*, *Theileria*, Cattle, Goats, Sheep, Red deer, Ruminants, Slovakia

## Abstract

Some piroplasmids and haemotropic mycoplasmas are important pathogens affecting domestic and wild animals, leading to various clinical symptoms and economic losses. This study aimed to determine, for the first time, the prevalence of haemotropic mycoplasmas and *Babesia*/*Theileria* species in domestic and wild ruminants in Slovakia, Central Europe. Blood samples from cattle, goats, and sheep (*n* = 549) and liver samples from red deer (*n* = 43) were collected across Slovakia between 2008 and 2024. DNA was extracted and screened by PCR and sequencing for haemotropic mycoplasmas and piroplasmids. The overall prevalence of haemotropic mycoplasmas was highest in cattle at 53.3% (124/232) and in sheep at 60% (90/150), whereas the prevalence in goats was substantially lower (4.2%; 7/167). Specifically, *Mycoplasma wenyonii* and “*Candidatus* Mycoplasma haematobovis” were detected in cattle, while *Mycoplasma ovis*-like microorganisms were identified in sheep and goats. For *Babesia*/*Theileria* species, a prevalence of 1.8% was recorded in goats, with no detections in sheep, and a prevalence of 65.1% was confirmed in red deer, where sequencing confirmed the presence of *Theileria capreoli*. This study presents the first report on the prevalence of haemotropic mycoplasmas in ruminants in Slovakia, highlighting the need for further research into transmission dynamics and potential zoonotic risks.

## Highlights

• First study on haemotropic *Mycoplasma* spp. in ruminants in Slovakia (60% in sheep, 53.3% in cattle, and 4.2% in goats).
• *Mycoplasma wenyonii* and “*Candidatus* Mycoplasma haematobovis” detected in cattle.
• *Mycoplasma ovis*-like microorganisms identified in sheep and goats.
• High *Theileria* spp. prevalence in red deer (65.1%), absent in sheep; *T. capreoli* confirmed by sequencing.

## Graphical abstract

**Figure:**
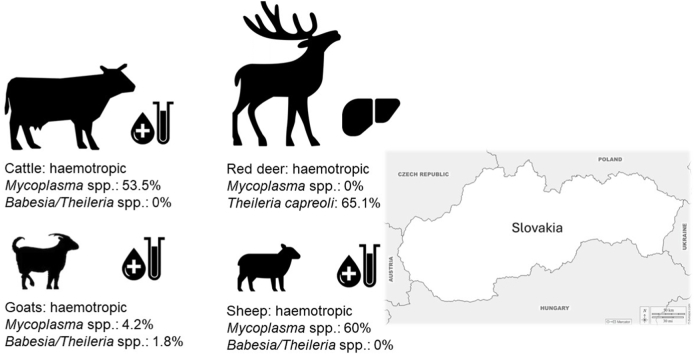

## 1 Introduction

Piroplasmids (order Piroplasmida, phylum Apicomplexa) are protozoan parasites that include the genera *Babesia*, *Theileria*, and *Cytauxzoon*. These parasites primarily infect the red blood cells and/or leukocytes of vertebrate hosts (Florin-Christensen et al., 2023). Among domestic mammals, ruminants are particularly affected by piroplasmid infections, which often lead to significant clinical and economic consequences.

Infections caused by *Babesia* spp. and *Theileria* spp. in ruminants can range from subclinical to severe. Infections in indigenous or wild hosts are often asymptomatic due to a long-standing host-parasite coevolution. However, in domestic cattle, sheep, and goats, particularly in non-endemic regions or in breeds without prior exposure, piroplasmids may cause acute disease characterized by fever, haemolytic anaemia, jaundice, anorexia, and reduced productivity. Severe cases can progress to complications such as abortion, neurological signs, respiratory distress, or death, depending on the species involved and the hostʼs immune status (Kiara et al., 2018; Kumar et al., 2021; Schnittger et al., 2022). Transmission of these parasites occurs *via* ixodid ticks, with vector specificity largely dependent on the parasite species and geographic region. In Slovakia, the presence of *Theileria* spp. in cervids has already been documented, highlighting the role of wild ungulates as potential reservoirs of piroplasmid infections. (Kazimírová et al. (2018) found *Theileria* spp. in red deer (*Cervus elaphus*) and roe deer (*Capreolus capreolus*), indicating their circulation among natural hosts and potential transmission by *I. ricinus* ticks. More recently, they expanded on this research, revealing a high genetic diversity of *Theileria* strains in cervids (Kazimírová et al., 2024).

Haemotropic mycoplasmas are gram-negative bacteria that infect red blood cells in various domestic and wild animals worldwide. Previously classified as *Haemobartonella* and *Eperythrozoon* species, they have been reclassified to the genus *Mycoplasma* based on 16S rRNA and RNase P gene analyses, as well as their morphological similarities (Neimark et al., 2001; Peters et al., 2008).

The epidemiology and transmission of haemotropic mycoplasmas remain poorly understood. It is assumed that transmission occurs through blood contact, either iatrogenically or *via* animal fighting. Additionally, various blood-feeding arthropods, including ticks, mosquitoes, lice, and certain flies (e.g. Muscidae and Tabanidae), have been considered as potential mechanical vectors (reviewed by Arendt et al., 2024). Transplacental transmission has also been demonstrated in cattle (Hornok et al., 2011; Sasaoka et al., 2015; Girotto-Soares et al., 2016; Niethammer et al., 2018; de Souza Ferreira et al., 2024).

Two haemotropic *Mycoplasma* species, *Mycoplasma wenyonii* and “*Candidatus* Mycoplasma haematobovis”, have been identified in cattle, both of which are distributed worldwide (de Souza Ferreira et al., 2024). Most infections are subclinical (Messick, 2004; de Souza Ferreira et al., 2024), but in stressed herds or when co-infected with *Anaplasma* spp. or *Babesia* spp., acute and severe disease may occur, potentially leading to death (Hofmann-Lehmann et al., 2004). Clinical signs of bovine mycohaemoplasmosis include anaemia, anorexia, diarrhoea, depression, transient fever, lymphadenopathy, swollen teats and scrotum, limb oedema, poor coat condition, weight loss, infertility, reproductive failure, decreased milk production, and low birth weight of calf (Smith et al., 1990; Montes et al., 1994; Messick, 2004).

Other important haemotropic *Mycoplasma* species include *M. ovis* and “*Candidatus* Mycoplasma haemovis”. *Mycoplasma ovis* infects various domestic and wild ruminants, including sheep, goats, white-tailed deer, and reindeer, and has even been detected in humans (Neimark et al., 2004; Stoffregen et al., 2006; Boes et al., 2012; Hornok et al., 2012; Maggi et al., 2013).

Clinical manifestations range from mild to severe and may include hemolytic anaemia, icterus, and depression, along with poor weight gain. The disease can cause mortality in lambs and in young adult sheep; however, deaths in adult sheep are uncommon (Campbell et al., 1971). Chronic infections in older animals are usually asymptomatic or present with mild clinical signs and persistent bacteremia (Gulland et al., 1987; Mason and Statham, 1991).

*Mycoplasma ovis* has a global distribution, with a high prevalence reported in Australia, Brazil, Iraq, China, Malaysia, Mexico, and Turkey (reviewed by Paul et al., 2020). However, the actual distribution remains unknown due to limited epidemiological data on ovine haemoplasmosis.

The importance of these investigations is underscored by the fact that certain haemoplasma species have been isolated from human patients (Pires dos Santos et al., 2008). In Inner Mongolia, China, a high prevalence of haemotropic mycoplasmosis was reported, with 35.4% of 1529 individuals testing positive (Yang et al., 2000; Hu et al., 2009). Additionally, ovine haemoplasma has been isolated from a human clinical case (Sykes et al., 2010). In North America, *M. ovis* was detected in people with close animal and arthropod contact, including veterinarians and their families (Maggi et al., 2013). Despite this, the zoonotic potential of haemotropic *Mycoplasma* species remains poorly understood and may be largely overlooked.

There are limited data on the prevalence of haemotropic *Mycoplasma* species and piroplasmid infections in ruminants in Slovakia. To address this gap, the present study aimed to determine the prevalence of these pathogens in both domestic ruminants (cattle, sheep, and goats) and wild ungulates (red deer) throughout Slovakia.

## 2 Materials and methods

### 2.1 Sample collection

**Fig. 1:**
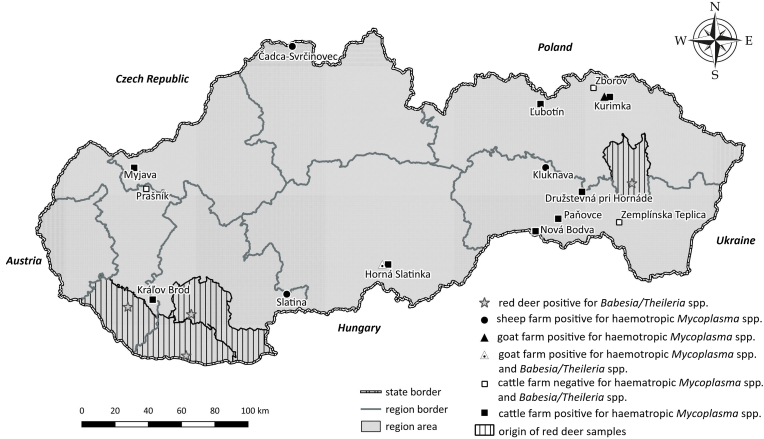
Map of Slovakia with sampling sites where individual animals (cattle, goats, sheep, and red deer) originated, as well as the locations of positive and negative farms. Symbols indicate the location of positive/negative farms. State borders, region borders, and regional areas are also indicated.

EDTA-treated blood samples were collected from cattle, goats, and sheep on 16 livestock farms across western, southern, and eastern Slovakia during the late spring to autumn months of 2008, 2016, 2018, and 2024 (Fig. 1). A total of 549 blood samples were analyzed, including 232 samples from cattle (*Bos taurus*), 167 samples from goats (*Capra aegagrus hircus*), and 150 samples from sheep (*Ovis aries*). The cattle were raised for milk and meat production, while the goats and sheep were primarily kept for milk production.

The animals were housed in stalls at night and grazed meadows and pastures during the day. While the cattle and sheep did not exhibit any clinical symptoms of infection, a few goats occasionally showed mild signs of apathy or lethargy. Additionally, liver samples from red deer (*Cervus elaphus*) were collected from the eastern district (Vranov nad Topľou, *n* = 25) and the neighboring southern districts (Dunajská Streda, Komárno, Nové Zámky, *n* = 18) of the country during the years 2022 and 2023, respectively (Fig. 1). The blood and liver collections were conducted following ethical standards and legal regulations of the Slovak Republic.

### 2.2 DNA extraction and PCR

Genomic DNA was extracted from EDTA-treated blood and liver samples using the DNeasy Blood and Tissue Kit (Qiagen, Hilden, Germany) according to the manufacturer's instructions. All extracted DNA samples were eluted in 50 μl of nuclease-free water and stored at −20 °C until PCR analyses.

PCR amplification of the 12S rRNA gene of vertebrates was conducted for each sample to serve as a quality control measure for the extracted tissue DNA (Humair et al., 2007). Only the samples that tested positive were subjected to further screening for pathogens. Genomic DNA samples were screened for the presence of haemotropic *Mycoplasma* spp., using the genus-specific primers Myco322s and Myco938as, targeting a 600-bp region of the 16S rRNA gene (Varanat et al., 2011). *Babesia*/*Theileria* spp. was detected using a PCR protocol targeting the 18S rRNA gene (Casati et al., 2006). PCR reactions were performed using MasterTaq DNA Polymerase (Eppendorf AG, Hamburg, Germany). In each PCR assay, a positive control with previously sequenced DNA and a negative control containing nuclease-free water were used. Ten percent of the positive PCR products targeting haemotropic mycoplasmas and all samples positive for *Babesia*/*Theileria* spp. were purified using the NucleoSpin® Gel and PCR Clean-up Kit (Macherey-Nagel GmbH & Co., Düren, Germany), and subsequently sequenced bidirectionally by Sanger sequencing with the same primers used for the PCR amplifications.

### 2.3 Sequencing and phylogenetic analysis

The nucleotide sequences of *Mycoplasma* spp. were manually edited in MEGA 11 and compared with GenBank entries by BLAST (Tamura et al., 2021). For the alignment of the homologous nucleotide sequences, the ClustalW program was used.

The nucleotide sequences obtained in this study were deposited in the GenBank database under the following accession numbers: 16S rRNA gene of “*Candidatus* M. haematobovis” (PQ728987, PQ728988), 16S rRNA gene of *M. wenyonii* (PQ738962-PQ738967), 16S rRNA gene of *M. ovis* (PQ776912-PQ776915), 18S rRNA gene of *Theileria* spp. (PQ682422-PQ682427).

### 2.4 Statistical analysis

Fisher's exact test was used to compare the prevalence of mycoplasmas and piroplasmids among domestic ruminants (cattle, sheep, goats). All results were considered statistically significant at the *P* < 0.05 level. DNA samples of reed deer were not included in the statistical analysis due to a different type of sample (liver *vs* blood) used for DNA extraction.

## 3 Results

The overall prevalence of haemotropic *Mycoplasma* spp. was 53.3% (124/232) in cattle, 4.2% (7/167) in goats, and 60% (90/150) in sheep (Table 1). Cattle from three farms, Prašník, Zborov, and Zemplínska Teplica, were negative for haemotropic *Mycoplasma*. Among the cattle farms where infections were detected, the prevalence ranged from 11.1% in Nová Bodva to 83.3% in Horná Slatinka. In sheep farms, the prevalence ranged from 24.5% to 80.4%, while in goat farms, it was 3.8% in Horná Slatinka and 10% in Kurimka (Table 1).

**Table 1:** Prelevance of haemotropic *Mycoplasma* spp. and *Babesia/Theileria* spp. in domestic and wild ruminants in Slovakia.

Farm/Locality	Year	*Mycoplasma* spp. positive/examined (prevalence)	*Babesia*/*Theileria* spp. positive/examined (prevalence)
**Cattle**			
Ľubotín	2008	32/40 (80.0%)	0/40
Kráľov Brod	2016	15/22 (68.2%)	0/22
Kurimka	2016, 2018	35/60 (58.3%)	0/60
Horná Slatinka	2016	5/6 (83.3%)	0/6
Prašník	2016	0/6	0/6
Zborov	2016	0/18	0/18
Paňovce	2016	7/17 (41.2%)	0/17
Družstevná pri Hornáde	2016	10/13 (76.9%)	0/13
Nová Bodva	2016	2/18 (11.1%)	0/18
Zemplínska Teplica	2016	0/9	0/9
Myjava	2024	18/23 (78.3%)	0/23
Total		124/232 (53.5%)	0/232
**Goats**			
Horná Slatinka	2016	6/157 (3.8%)	3/157 (1.9%)
Kurimka	2016	1/10 (10.0%)	0/10
Total		7/167 (4.2%)	3/167 (1.8%)
**Sheep**			
Čadca-Svrčinovec	2023	41/51 (80.4%)	0/51
Slatina	2023	37/50 (74.0%)	0/50
Kluknava	2023	12/49 (24.5%)	0/49
Total		90/150 (60.0%)	0/150
**Red deer**			
Vranov nad Topľou	2022	0/25	13/25 (52.0%)
Dunajská Streda, Komárno, Nové Zámky	2023	0/18	15/18 (83.3%)
Total		0/43	28/43 (65.1%)

Sequencing of the 16S rRNA gene identified “*Candidatus* M. haematobovis” and/or *M. wenyonii* in cattle samples, while *M. ovis* was detected in sheep and goat samples. The 16S rRNA gene sequences of “*Candidatus* M. haematobovis” isolates from our study showed 99.49–100% identity with 16S rRNA gene sequences of *Mycoplasma* isolates from e.g. cattle, water buffaloes, sheep, goats, dogs, and/or cats from elsewhere in the world (e.g. Cuba, China). *Mycoplasma wenyonii* 16S rRNA gene sequences were 97.52–100% identical with those of isolates from cattle, and small ruminants identified in Cuba, India, South Korea, Turkey, and other countries, and 97.67–99.83% identical with the 16S rRNA gene identified in the genome of *M. wenyonii* Massachusetts strain (GenBank: CP003703). The sequences of *M. ovis* from our study were 99.5–100% identical with sequences obtained from sheep and/or goats from Hungary, Brazil, China, and Japan. They also showed 99.83% identity with 16S rRNA gene sequences of TX1294-C and TX1294-D isolates from human patients diagnosed with *M. ovis* infection in the USA (GenBank: GU230143-GU230144) and 99.5% identity with the 16S rRNA gene locus reported from the genome of the *M. ovis* Michigan strain (GenBank: CP006935). Cattle and sheep showed significantly higher prevalence rates of haemotropic mycoplasmas than goats (*P* < 0.00001).

All red deer samples tested negative for haemotropic *Mycoplasma* spp. *Babesia*/*Theileria* spp. were detected in three goats from the Horná Slatinka farm, although the sequencing of the PCR products did not yield a readable chromatogram.

The overall prevalence of *Theileria* sp. in red deer was 65.1% (28/43), with a higher prevalence in animals from southern Slovakia (Dunajská Streda, Komárno, Nové Zámky; 83.3%, 15/18) compared to those from eastern Slovakia (Vranov nad Topľou; 52.0%, 13/25). Sequencing of the positive amplicons confirmed the presence of *T. capreoli* (GenBank: PQ682422-PQ682427), which were identical to each other and showed 100% identity with isolates, e.g. from *H. concinna* ticks previously obtained in Slovakia (GenBank: PP086528) or the *T. capreoli* GP-B13 isolate from deer ked (*L. fortisetosa*) from Poland (GenBank: MW531681).

## 4 Discussion

Haemotropic *Mycoplasma* infections in cattle remain understudied. In Europe, existing research has focused on countries such as Switzerland, Germany, France, England, Hungary, Sweden, and Bosnia and Herzegovina (Hofmann-Lehmann et al., 2004; Meli et al., 2010; Hoelzle et al., 2011; Ayling et al., 2012; Hornok et al., 2011, 2012; Ade et al., 2018; Niethammer et al., 2018; Nouvel et al., 2019; Stevanović et al., 2020; Waller et al., 2023). Studies have also been conducted in other regions, including Asia, Africa, and the Americas (Tagawa et al., 2008, 2010, Tagawa et al., 2012; Nishizawa et al., 2010; Su et al., 2010; Fujihara et al., 2011; Girotto et al., 2012; Song et al., 2013; Girotto-Soares et al., 2016; Byamukama et al., 2020; Schambow et al., 2021; Erol et al., 2023). In Europe, the prevalence of haemotropic mycoplasmas in domestic cattle is notably high, ranging from 50% to 97% (de Souza Ferreira et al., 2024). In the USA, a study conducted in 64 herds in Wisconsin and 18 herds in Michigan found a 100% herd prevalence (Schambow et al., 2021).

Our study reports a high prevalence of haemotropic *Mycoplasma* infections in cattle across Slovakia, with positive detections on 8 out of 11 farms. The prevalence rates ranged from 11.1% to 83.3%, with an overall prevalence of 53.3%, underscoring the widespread nature of these infections in this region. Sequencing of the positive samples confirmed the presence of *M. wenyonii* and “*Candidatus* M. haematobovis”. Interestingly, three farms in our study were free of haemotropic *Mycoplasma*, which raises questions about the factors influencing transmission. Previous studies have suggested environmental or vector-borne components in the transmission of *M. wenyonii*, with Kim et al. (2024) highlighting significantly higher infection rates in grazing cattle than housed cattle. This seasonal and environmental variation was also seen in other studies, where most infections were detected during late summer and early winter (Strugnell and McAuliffe, 2012). These findings point to the potential role of environmental factors, such as vector activity, in shaping infection dynamics.

Noteworthy observation in our study was the absence of piroplasmids in cattle blood samples. Tagawa et al. (2013) suggested that an infection with haemotropic mycoplasmas may inhibit or even prevent a subsequent infection with piroplasmids and referred to this as a phenomenon of interference. In their study, cattle infected with haemotropic mycoplasmas tended to resist infection with *Theileria orientalis.* This hypothesis is supported by similar observations in water buffaloes (*Bubalus bubalis*, family Bovidae) from the grassland natural reserve in Mórahalom, where high prevalence rates of *M. wenyonii* (91.2%) and “*Candidatus M. haematobovis”* (73.3%) were coupled with a lack of *Theileria* spp., *Babesia* spp., and *Anaplasma phagocytophilum* infections (Hornok et al., 2017). These findings suggest that the immune response to one pathogen may offer some degree of resistance to another.

As mentioned earlier, our study did not detect piroplasmids in any cattle blood samples, regardless of whether the animals were positive or negative for haemotropic mycoplasmas. This suggests that piroplasmids may have a low prevalence or were absent in the cattle populations from the regions investigated during the sampling periods. Additionally, there may be pathogen interference between haemotropic mycoplasmas and piroplasmids, or the absence of piroplasmids could be influenced by environmental, ecological, or vector-related factors.

In the livers of red deer, we did not detect any haemotropic mycoplasmas. Previous research indicated that the prevalence of haemotropic mycoplasmas in white-tailed deer (*Odocoileus virginianus*) in North Carolina is 89% (Maggi et al., 2013), while in sika deer (*Cervus nippon*) in Japan, it was only 9% (Watanabe et al., 2010). In Europe, data on the prevalence of haemotropic mycoplasmas in wild ungulates are limited to two studies, one conducted in Hungary (Hornok et al., 2018) and another in Sweden (Waller et al., 2023). Waller et al. (2023) analyzed the spleens of 15 red deer, 11 fallow deer, 4 roe deer, and 10 wild boars, finding a zero prevalence for *M. wenyonii*. In the study of Hornok et al. (2018), the prevalence varied by animal species, with water buffalos exhibiting the highest rates, 73.3% for “*Candidatus* M. haematobovis” and 91.2% for *M. wenyonii*. Conversely, the lowest prevalence was observed in mouflon blood samples, with zero prevalence for “*Candidatus* M. haematobovis” and 6.3% for *M. wenyonii*. Additionally, the prevalence was higher in blood samples compared to spleen samples. In red deer, the prevalence in blood samples was 64.6% for *M. wenyonii* and 45.8% for “*Candidatus* M. haematobovis”, while only 25% of spleen samples from red deer were positive for *M. wenyonii* and 4.2% for “*Candidatus* M. haematobovis” (Hornok et al., 2018). The prevalence of haemoplasmas was significantly higher in water buffalos compared to red deer, fallow deer, and roe deer, all of which were from the same region (the grassland natural reserve at Mórahalom) (Hornok et al., 2018). In contrast, our study found that 65.1% of red deer tested positive for piroplasmids, with all sequenced samples identified as *T. capreoli*. High infection rates of *Theileria* spp. were also detected in ungulates (red deer, roe deer, and fallow deer) across Europe (Sawczuk et al., 2008; Skotarczak et al., 2008; Galuppi et al., 2011; Fuehrer et al., 2013; Hornok et al., 2017) and in a recent study from Slovakia (Kazimírová et al., 2018), supporting their role as reservoirs for the pathogen. Although the liver is not the primary site of *Mycoplasma* infection, previous studies (e.g. Aktas and Ozubek, 2017) have demonstrated that *Mycoplasma* DNA can be detected in liver tissues due to liver vascularization and the possibility of residual infected erythrocytes being present. Thus, the zero prevalence for haemoplasmas observed in red deer livers in our study may not be strictly attributed to the type of sample used (liver and not blood) but also could be the result of the previously mentioned interference phenomenon; in this case, the infection with *Theileria* spp. (or possibly other pathogens not screened in our study) could protect animals from infection with haemotropic mycoplasmas. Unfortunately, comparable data specifically on the detection rates of haemotropic mycoplasmas in red deer or other cervids in Central Europe are scarce. Most available studies focus either on domestic ruminants or on a few isolated cases in wildlife, often using different tissues (e.g. blood *vs* spleen) and methodologies, making direct comparisons challenging.

In our study, we observed a relatively low prevalence of haemotropic *Mycoplasma* in goats (4.2%), which contrasts sharply with the much higher prevalence observed in sheep (60%). Similar findings were reported by Aktas and Ozubek (2017) in Turkey, where goats had a prevalence of 6.2%, compared to 11.3% in sheep. This suggests that sheep might be more susceptible to *M. ovis*, which could be related to species-specific differences in immune responses or vector exposure. In Europe, research on *M. ovis* is limited, with a notable study conducted in Hungary (Hornok et al., 2009). Additionally, one European wildcat in Germany was found positive for *M. ovis* (Unterköfler et al., 2022). Although we did not detect piroplasmids in sheep, 1.8% of goats tested positive for piroplasmids, though sequencing results were inconclusive. The absence of detectable piroplasmids in sheep is consistent with studies indicating that sheep may not be as commonly involved in the transmission of piroplasmids compared to other livestock (e.g. cattle and goats). Sheep farming in Slovakia has a long tradition and is well developed, which means that a significant number of people may be exposed to this pathogen. To date, there are no confirmed cases of haemotropic *Mycoplasma* infections in humans reported in Europe. Although zoonotic transmission remains theoretically possible, particularly for immunocompromised individuals in close contact with infected animals.

## 5 Conclusions

To the best of our knowledge, this is the first study of haemotropic mycoplasmas in both domestic and wild ruminants in Slovakia, Central Europe. Our findings indicate a high prevalence of haemoplasmas in cattle and sheep farms, while goat farms showed a low prevalence. None of the red deer liver samples tested positive for haemoplasma, but we detected a high prevalence of piroplasmids, identified by sequencing as *T. capreoli*. This finding supports the hypothesis that red deer may serve as a reservoir for the pathogen. In Slovakia, farmers commonly graze cattle, sheep, and goats, which poses a high risk for the transmission of vector-borne pathogens. Further research is required to investigate the specific risk factors contributing to the increased prevalence of haemoplasma infections.

## Statement on the use of AI-assisted technologies

During the preparation of this article, the authors used Grammarly (https://www.grammarly.com/) to correct grammatical errors and improve readability. After using this tool, the authors reviewed and edited the content as needed. The authors take full responsibility for the content of the published article.

## CRediT authorship contribution statement

**Dana Zubriková:** Conceptualization, Writing – original draft, Investigation, Methodology. **Lucia Vargová:** Investigation, Methodology. **Júlia Halapy:** Investigation, Writing – review & editing. **Branislav Lukáč:** Investigation, Writing – review & editing. **Veronika Blažeková:** Investigation, Methodology, Writing – review & editing. **Klaudia Mária Švirlochová:** Investigation, Methodology, Writing – review & editing. **Eva Čisovská Bazsalovicsová:** Investigation, Writing – review & editing. **Ján Čurlík:** Investigation, Writing – review & editing. **Ivana Heglasová:** Investigation, Writing – review & editing. **Bronislava Víchová:** Writing – review & editing, Supervision, Methodology, Project administration, Funding acquisition.

## Ethical approval

Manipulation with animals during the blood taking as well as handling of the carcasses were conducted following the authorization by the Ministry of Environment of the Slovak Republic under permit No. 498/2018-6.3 and samples were further transported to the Laboratory of Molecular Ecology of Vectors (Institute of Parasitology SAS).

## Funding

The study has been financially supported by the projects 10.13039/501100006109VEGA 2/0051/24, 10.13039/501100006109VEGA 2/0033/25, 10.13039/501100005357APVV SK-10.13039/501100022871SRB 23–0046, and 10.13039/501100005357APVV 21–0166.

## Declaration of competing interests

The authors declare no competing financial interests or personal relationships that could have appeared to influence the work reported in this paper.

## Data Availability

The data supporting the conclusions of this article are included within the article.
